# A differential diagnosis method for systemic CAEBV and the prospect of EBV-related immune cell markers via flow cytometry

**DOI:** 10.1080/07853890.2024.2329136

**Published:** 2024-03-19

**Authors:** Jie Jin, Xia Mao, Donghua Zhang

**Affiliations:** Department of Hematology, Tongji Hospital, Tongji Medical College, Huazhong University of Science and Technology, Wuhan, Hubei, China

## Abstract

Chronic active Epstein-Barr virus (CAEBV) infection of the T-cell or Natural killer (NK)-cell type, systemic form (systemic CAEBV or sCAEBV) was defined by the WHO in 2017 as an EBV-related lymphoproliferative disorder and is listed as an EBV-positive T-cell and NK-cell proliferation. The clinical manifestations and prognoses are heterogeneous. This makes systemic CAEBV indistinguishable from other EBV-positive T-cell and NK-cell proliferations. Early diagnosis of systemic CAEBV and early hematopoietic stem cell transplantation can improve patient prognosis. At present, the diagnosis of systemic CAEBV relies mainly on age, clinical manifestations, and cell lineage, incurring missed diagnosis, misdiagnosis, long diagnosis time, and inability to identify high-risk systemic CAEBV early. The diagnostic methods for systemic CAEBV are complicated and lack systematic description. The recent development of diagnostic procedures, including molecular biological and immunological techniques such as flow cytometry, has provided us with the ability to better understand the proliferation of other EBV-positive T cells and NK cells, but there is no definitive review of their value in diagnosing systemic CAEBV. This article summarizes the recent progress in systemic CAEBV differential diagnosis and the prospects of flow cytometry.

## 1. Introduction

Epstein–Barr virus (EBV) infection occurs in 90% of adults, and primary EBV infection occurs through naive B cells and establishes a latent infection. An impaired balance between the host immune response and EBV replication can lead to a variety of EBV-positive lymphoproliferative diseases involving B, T, or NK cells. Infectious monocytosis (IM) is caused by infection of B cells and is self-limiting [1]. In a small number of people, due to genetic predisposition and EBV infection [2,3], NK/T cells continue to be infected, which can cause EBV-associated lymphoproliferative diseases dominated by NK/T cells, including systemic CAEBV [4,5]. In Asia, most CAEBV cases involve T/NK cells [6]. EBV-infected B cells have been reported in America [7]. Currently, the term systemic CAEBV mainly refers to a disease of T or NK cell origin [8–10].

Systemic CAEBV needs to be differentiated from other EBV-positive T-cell and NK-cell proliferations in the revised 2017 WHO classification, including (1) EBV-positive hemophagocytic lymphohistiocytosis (EBV + HLH); (2) systemic CAEBV; (3) cutaneous CAEBV, hydroa vacciniforme-like lymphoproliferative disorder(HV-LPD); 4) cutaneous CAEBV, severe mosquito bite allergy (sMBA); (5) systemic EBV-positive T-cell lymphoproliferative disease of childhood (sEBV(+)TCL); (6) aggressive NK-cell leukemia (ANKL); (7) extranodal NK/T-cell lymphoma, nasal type (ENKTL); (8) nodal peripheral T-cell lymphoma, EBV-positive (EBV + PTCL, NOS). Many use EBV ± T/NK-LPDs to broadly refer to EBV + T-cell and NK-cell proliferation. According to the 2022 International Consensus Classification (2022 ICC), chronic active EBV disease replaces chronic active EBV infection [10,11]. HV-LPD and sMBA are distinguishable by cutaneous symptoms, so they are beyond the scope of our discussion. Although chronic lymphoproliferative disorder of NK cells (CLPD-NK) involves clonal proliferation of NK cells [12], this process is not related to EBV infection and is not discussed in this article.

The prognosis of systemic CAEBV is heterogeneous, ranging from asymptomatic to death within weeks. Systemic CAEBV can transform into two deadly diseases—HLH and chemotherapy-resistant tumors. Sixteen percent of systemic CAEBV patients progress to NK/T-cell lymphoma or aggressive NK-cell leukemia [13]. Approximately 24 ∼ 58% of CAEBV patients met the HLH criteria in selected studies [14,15]. The earlier the treatment is, the better the survival [16]. However, the disease eventually relapses [17]. Current treatment strategies are not effective. Allogeneic hematopoietic stem cell transplantation (allo-HSCT) is the only curative treatment for this disease [16,18,19], and how to choose the appropriate timing for allo-HSCT is still unknown. It is important to recognize this phenomenon in the early stages before progression.

How can the early diagnosis of systemic CAEBV be improved? It is possible to improve the diagnosis of NK/T cells by using flow cytometry to examine the immunophenotypic characteristics of NK/T cells in patients with systemic CAEBV. To diagnose systemic CAEBV, it is necessary to make a comprehensive judgment based on clinical manifestations and various laboratory tests. Several reviews [4,20] have analyzed several factors that hinder the separation of clinical risk groups: (1) varied manifestations, (2) nonspecific and overlapping histopathological and immunophenotypic features and failure to predict clinical behavior, (3) methods for detecting infected cells that have not been widely recognized, 4) molecular clonality that is not necessarily an indication, and (5) an association with HLH. These problems all point to the current deficiencies in diagnostic parameters. In recent years, studies of EBV-positive T-cell and NK-cell proliferation have made progress in terms of flow cytometry, EBV-DNA, etc. This article will assess valuable differential diagnosis method and EBV-related immune cell markers *via* Flow Cytometry (FCM).

### 1.1. Systemic CAEBV’s current differential diagnosis

#### 1.1.1. Diagnostic criteria and evaluation

There are now two versions of the accepted diagnostic criteria, which are from the WHO 2017 Classification and the research group Measures Against Intractable Diseases of the Ministry of Health, Labor and Welfare of Japan (MHLW research group) [21]. The differences between the diagnostic criteria of Japan and the WHO 2017 Classification lie in several aspects [21]. First, the WHO 2017 Classification clarifies the types of EBV-infected cells in its definition, not the criteria; 2022 ICC requires that CAEBV consists of only T and NK cells [10]. Second, according to the 2017 WHO Classification, methods are needed to identify EBV RNA or viral proteins using pathological specimens. In addition, the criteria of the MHLW research group require EBV infection of T or NK cells in the affected tissues or the PB without restriction on methods or specimens. Third, HV-LPD and sMBA were not diagnosed as CAEBV according to the MHLW research group. In addition to diagnostic criteria differences, there is a lack of clarity in some respects, leading to diagnostic difficulty [20]. For example, (1) IM symptoms include many subsymptoms and are not specific. Patients may demonstrate any combination of these clinical manifestations, and atypical scenarios beyond IM symptoms are not uncommon. Although IM-like symptoms should be fulfilled, in some studies, this condition was considered to indicate an active state [22]. (2) There is a diversity of infected cell types; for example, some patients may be infected with B cells at the same time, or the cell types involved in bloodstream infections and tissue infections might be heterogeneous. (3) Patients with known immunodeficiency need time to be excluded, and therapeutic intervention is often delayed. Systemic CAEBV can be diagnosed first, but systemic CAEBV can be diagnosed even if EBV + lymphoma subsequently develops; if EBV + lymphoma is diagnosed early, systemic CAEBV cannot be diagnosed. However, there is still a gray zone in the diagnosis of some EBV + lymphomas and known immunodeficiencies. Case reports of CAEBV with ‘autoimmune disorder’, such as ‘Crohn’s disease’ or ‘polymyositis’, have been published [23,24].

The diagnostic criteria for selected EBV-positive T/NK cell proliferation are listed in Table 1. The main differential diagnoses of NK and T-cell lymphoproliferation differ [33]. The diagnostic criteria for CAEBV are based on clinical and laboratory criteria. However, the diagnosis of sEBV(+)TCL, ANKL, ENKTL, and NPTCL relies mainly on the histopathological immunophenotype. Although it is difficult for pathological specimens to be obtained from patients with systemic CAEBV, it is also necessary to obtain specimens as much as possible to exclude other tumors. Notably, EBV-HLH is not broadly HLH but rather EBV-positive. Differential diagnosis of EBV-HLH, CAEBV combined with HLH, neoplasia-associated HLH (EBV+), and familial HLH (EBV+) is essential. Because their treatment strategies are different. The diagnosis of HLH is based on clinical manifestations and laboratory findings. If the criteria are met, the patient can be diagnosed regardless of cause. The clinical manifestation of HLH can be the initial clinical manifestation of CAEBV or any EBV + T/NK-cell lymphoma, which can be classified as neoplasia-associated HLH^34^. Neoplasia-associated HLH is dismal and often related to poor prognosis. In contrast, EBV-HLH is considered benign and likely self-limiting. Aggressive treatment with multiagent chemotherapy and allo-HSCT can lead to overtreatment and a significant risk for patients who ‘simply’ have nonneoplastic EBV-HLH. Clinical differentiation between nonneoplastic EBV-HLH and neoplasia-associated HLH is exceedingly difficult. When EBV-HLH fails to respond to HLH-directed therapy, one must consider that an underlying diagnosis of neoplasia-associated HLH is present. Fortunately, the processes of identifying the cause of HLH and diagnosing CAEBV are not contradictory.

**Table 1.** Diagnostic criteria for EBV-positive T/NK cell proliferation.

Disease	Criteria	Criteria/Supplement	Reference
Sys CAEBV	Include all the following conditions from (1) to (4): (1) IM-like symptoms persisting for > 3 months (2) Increased EBV-DNA (>10^2.5 copies/µg) in PB (3) Histological evidence of organ disease (4) Demonstration of EBV RNA or viral protein in affected tissues in patients without known immunodeficiency, malignancy, or autoimmune disorders	MHLW research group of Japan: (1) Sustained or recurrent IM-like symptoms persist for more than 3 months (2) Elevated EBV genome load in PB or the tissue lesion (3) EBV infection of T or NK cells in the affected tissues or the PB (4) Exclusion of other possible diagnoses: primary infection of EBV (infectious mononucleosis), autoimmune diseases, congenital immunodeficiencies, HIV, and other immunodeficiencies requiring immunosuppressive therapies or underlying diseases with potential immunosuppression. Patients who fulfilled criteria (1)–(4) were diagnosed with CAEBV	[15,21]
EBV-HLH	(1) Clinical criteria (fever and splenomegaly) (2) Laboratory criteria (cytopenia affecting 2 of 3 lineages in the peripheral blood, hypertriglyceridemia, and/or hypofibrinogenemia) (3) Histological criteria (hemophagocytosis in the BM, spleen, or lymph nodes)	Exclusion of Congenital immunodeficiency including familial HLH, neoplasia-associated HLH	[6,25]
sEBV(+)TCL	(1) Illness or symptoms including fever, persistent hepatitis, lymphadenopathy, hepatosplenomegaly, hemophagocytosis, and interstitial pneumonia (2) Can occur shortly after primary EBV infection or in the setting of CAEBV (3) Monoclonal expansion of EBV-infected T cells with an activated cytotoxic phenotype in tissues or peripheral blood	(1) Progressive clinical course with features of HLH (2) Overt infiltrates of CD8+ and CD56 − cytotoxic T-lymphocytes with small or occasionally medium to large-sized or large nuclei (3) Overt lymphoma or rearrangements of T-cell receptor (TCR) genes	[15,26]
ANKL	(1) Cells with a slightly immature-looking morphology with broad, pale cytoplasm and azurophilic granules, a slightly fine nuclear chromatin, and the occasional presence of nucleoli (2) sCD3–, CD56+, CD16, and CD57– immunophenotype (3) Genetically, germline configuration TCR- and IgH genes (4) Functionally, a high or sometimes absent non-major histocompatibility complex–restricted cytotoxicity.		[27–29]
ENKTL	(1) Vascular damage and destruction, prominent necrosis (2) Cytotoxic phenotype (3) Association with EBV	Histopathological diagnosis: CD56, EBER, and cytotoxic molecules are positive	[30,31]
EBV + PTCL, NOS	(1) A T follicular helper (TFH) cell phenotype, as defined by the expression of at least two (ideally three) of the following markers: CD10, BCL6, PD1, CXCL13, CXCR5, ICOS, and SAP (2) Exclusion from any of the specifically defined entities of mature T-cell lymphoma in the current classification	EBV+, included within the category of peripheral T-cell lymphoma, NOS.	[32]

**Figure 1.**
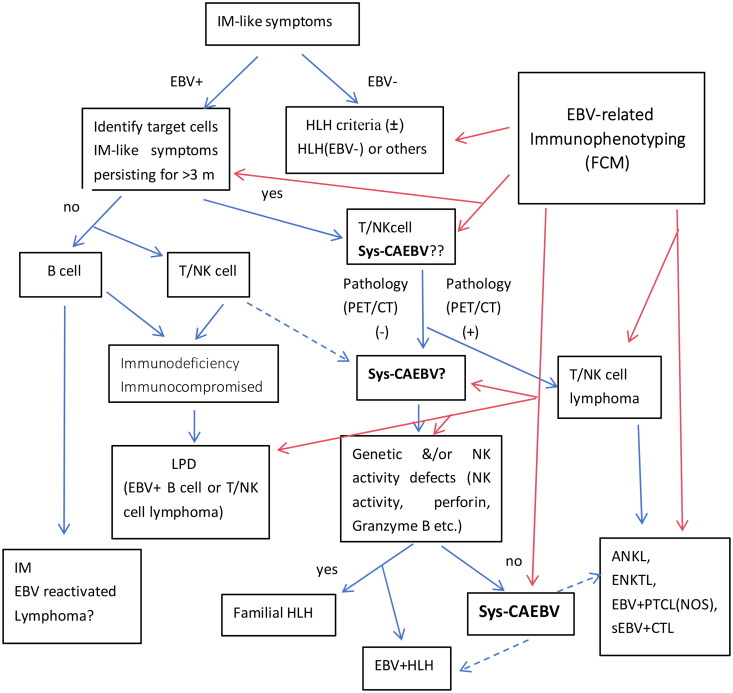
Proposed diagram illustrating the various approaches for diagnosing and managing EBV infection. Sys-CAEBV: systemic CAEBV, LPD: lymphoproliferative disorders, HLH: haemophagocytic lymphohistiocytosis, IM: Infectious monocytosis. FCM: flow cytometry Dotted arrow(): possible, red arrow () : reconfirmed.

Patients with an EBV genome load (+) in peripheral blood (PB) or an EBER (+) in tissue should be further examined. However, whether HLH occurs in combination should be evaluated first. Bone marrow or PB-related examinations, such as analyses of the function of NK cells, EBV DNA in plasma, and mutations, should also be considered to determine the risk of systemic CAEBV infection. Additionally, ongoing monitoring is needed. However, malignant transformation to T/NK malignancy needs to be confirmed. The diagnostic workup of patients with systemic CAEBV see Figure 1.

#### 1.1.2. Clinical features

Clinical findings can provide clues to the diagnosis of CAEBV. The complex mixture of tumorigenic processes of EBV-positive cells and physical symptoms of systemic inflammatory disease constitute the varied manifestations of systemic CAEBV. Except for a few skin symptoms and uveitis, the clinical symptoms of systemic CAEBV are not specific and cannot be distinguished from those of other EBV + T/NK proliferations.

Most of the reported cases were in Asia and Latin America. The disease was previously assigned to the pediatric and adult-onset patients are gradually being recognized. In 2008, a study in Korea revealed that CAEBV is the main cause of young adults’ EBV-associated T or NK cell malignancies [35]. According to a 2020 nationwide survey in Japan [19], half of the participants were adults. In 2021, a study in China reported that the median age was 33 years (range 14–83) in 19 adults [36]. Compared with pediatric-onset CAEBV, adult-onset systemic CAEBV has a poorer prognosis [37], and pediatric-onset CAEBV and adult-onset systemic CAEBV appear to be different disorders [19]. In most studies, the most common symptoms are recurrent fever, splenomegaly, hepatomegaly, lymphadenopathy, abnormal liver function, hemophilia, and HLH. The additional symptoms reported in a recent large-scale survey [19], in descending order of frequency, were cardiac dysfunction, aneurysm, gastrointestinal symptoms, neurological symptoms, vasculitis diagnosed by pathology, and uveitis. Life-threatening complications include HLH, coronary artery aneurism, hepatic failure, interstitial pneumonia, CNS involvement, gastrointestinal perforation, and myocarditis [15]. Rare complications, such as acute fibrinous and organizing pneumonia and refractory enteritis, have also been reported [38]. HLH or EBV + lymphoma often develop during the course of the disease [6]. Patients with T-cell type CAEBV present worse outcomes than those with NK-cell type CAEBV [39,40]. According to the findings of Kawamoto et al. lymphadenopathy was more common in patients with T-cell CAEBV, while skin involvement was more frequent in patients with NK-cell CAEBV. Some differences exist between different areas, including in the incidence of lymphadenopathy, interstitial pneumonitis, and cutaneous symptoms [17,41].

A comparison of the clinical manifestations of systemic CAEBV and other EBV-positive T/NK cell proliferation has been performed in several reviews [4,8,33,34,42–44]. These comparisons mainly cover aspects including areas with the highest prevalence, age group, clinical symptoms, location, clinical behavior (or prognosis) [8,33], and frequency of HLH^34^. All of these populations are more prevalent among Asians. Among the age groups, systemic CAEBV and sEBV(+)TCL patients had more pediatric-onset disease, while the others had more adult-onset disease. Among clinical symptoms, systemic CAEBV overlaps with most other CAEBV subtypes. Among them, ENKTL patients exhibit distinguishable presentations with symptoms of nasal obstruction or epistaxis. In 2021, a Chinese cohort [45] showed that the CAEBV group and EBV-associated lymphoma/leukemias group were not significantly different in age, sex, or common clinical manifestations. Systemic CAEBV, ANKL, and sEBV(+)TCL are systemic diseases. Although ENKTL and EBV(+) PTCL, NOS can also be widespread, they involve more typical sites. The anatomic location provides a very important clue for the diagnosis of some specific entities and is a critical diagnostic component for others, but by itself, it does not define any lymphoma type [46]. Clinically, systemic CAEBV presents more variability than other aggressive forms of CAEBV. ANKL and sEBV(+)TCL have more aggressive clinical courses. EBV-HLH seems to be the most benign. The frequencies of HLH, CAEBV, ANKL, and sEBV (+) TCL are often related to HLH. In 2022, Zheng et al. [22] found that the percentage of HLH is greater in ANKL than in CAEBV-NK and ENKTL. HLH is uncommon in patients with ENKTL and EBV (+) PTCL, NOS.

Theoretically, acute onset, atypical cytological features, and a highly aggressive clinical course are not features of systemic CAEBV. Severe cases of CAEBV with EBV-positive monoclonal T-cell proliferation were considered sEBV(+)TCL rather than CAEBV. There is still overlap between these entities, which cannot be separated only by clinical features. When we encounter patients with EBV infection whose general condition is poor and whose infection course has not reached 3 months, we cannot meet the recognized diagnostic criteria for systemic CAEBV. However, there is often a lack of strong evidence for the diagnosis of ANKL and ENKTL at that time. When a patient with persistent fever or inflammation is encountered in any department, the possibility of systemic CAEBV should be suspected, and further evaluation should be performed [47].

#### 1.1.3. Laboratory tests

##### 1.1.3.1. General test

General laboratory tests include routine blood tests, blood biochemical tests, coagulation function tests, cytokine analysis, ferritin, soluble CD25, and rheumatic and immunity-related tests. These tests are not required for diagnosis but reflect the basic status of patients. These tests are needed to detect an ‘active state’, determine the severity of inflammation, diagnose HLH, and identify high-risk patients and patients with poor outcomes. Thrombocytopenia and HLH are considered to be related to poor prognosis [40]. Liver dysfunction is a risk factor for mortality [6]. The levels of interleukin IL-6 and IL-10 were found to be independent prognostic factors in a cohort of children with CAEBV [48]. An EBV DNA concentration >10^5^ copies/mL, a platelet count <50 × 10^12^/L, an albumin concentration <30 g/L, and a serum ferritin concentration >5000 μg/L are risk factors for death in patients with CAEBV [49]. Disease activity was defined according to previous Japanese reports [6,19,50]. Patients with active disease were defined as those with persistent findings of inflammation, as follows: fever, liver dysfunction, progressive skin lesions, vasculitis, or uveitis, accompanied by a significant increase in EBV-DNA. Liver dysfunction was defined as an increase in alanine transaminase levels to 2 times higher than the upper limit of normal.

Laboratory tests reveal mainly cytopenia and abnormal liver function [15]. Dávila Saldaña, B. J [14]. reported that cytopenias are present in 80% of patients in their cohort. This decrease is reported mostly as a twofold or threefold reduction [36]. Kimura et al. [51] reported that the frequencies of liver dysfunction, thrombocytopenia, and anemia in these patients were 90%, 50%, and 48%, respectively. The levels of albumin, glutamic oxalacetic transaminase, alanine aminotransferase, triglycerides, and lactic dehydrogenase in peripheral blood were slightly increased [48]. IFN-γ, TNF-α, and IL-6 are increased in CAEBV, and the level is closely related to the severity of the disease [52,53]. Disseminated intravascular coagulation also occurs in a small portion of patients [36].

Generally, patients with ANKL presented with cytopenias, whereas patients with NKTCL tended to have normal blood cell counts. The frequency of HLH in ANKL patients was significantly greater than that in systemic CAEBV and ENKTL patients in a study by Zheng et al. [22].

The concrete level of cytopenia or liver dysfunction and comparisons with other diseases currently lack uniform descriptions. We could conclude that general laboratory test results for systemic CAEBV coincide with what HLH requires. It is necessary to check for HLH throughout the disease course using these tools, so these tests are basic.

##### 1.1.3.2. EBV status

Virology-related tests for systemic CAEBV, which include serological tests, EBV DNA in the blood (plasma/mononuclear cells) or body fluid, EBV DNA/RNA in specimens, EBV clonality and type, and EBV isolation, are indispensable [54]. However, the significance of some related content has changed in recent years. Other diseases are also associated with EBV, but the correlation varies. All the diseases discussed in this text except for ANKL are EBV positive [55]. The relationship between EBV and HLH is complex and wide-ranging. Among the various causes of nonspecific HLH, EBV is the most common infection. EBV + PTCL, NOS is an EBV + part of PTCL, NOS and the proportion of cases is unclear. EBV clonality, type, and isolation are usually tested in a research background and are rarely used clinically.

###### 1.1.3.3. EBV antibodies

In the past, serologic patterns of antibodies against the EBV protein were deemed part of the diagnostic criteria [54]. These included antiviral capsid antigen (VCA) IgG ≥ 1:5120, anti-early antigen (EA) IgG ≥ 1:640, and anti–Epstein–Barr nuclear antigen (EBNA) less than 1:2 [51]. The high false-positive rate for EBV-related antibody testing limits the effectiveness of disease diagnosis, and serological tests for EBV are now removed from the criteria. However, few studies have evaluated this topic.

High titers of VCA-IgG and EA-IgG are markers of increased EBV replication and thus are useful for the diagnosis of chronic active EBV infection [54]. It is important to note that EBV-specific antibody profiles can vary depending on individual differences in the host fluid immune response.

EBV serologic tests generally reveal high IgG antibody titers against EBV VCA (≥1:640) and EA (≥1:160) in each laboratory in patients with systemic CAEBV; positive IgA antibodies to VCA and/or EA are often demonstrated; one-third of the patients did not fulfill any of the above criteria [51].

Comparative studies of antibodies for different diseases are lacking, so the significance of differential diagnosis is limited. Low titers of serology-related antibodies have not been found to exclude CAEBV, as abnormally elevated (VCA-IgG ≥ 1:1520, anti-EA antibody ≥1:640) antibodies occur in only a small proportion of cases. In addition, there have been cases of negative EA-IgG antibodies whose EBV-DNA quantification is significantly increased.

###### 1.1.3.4. EBV DNA copies

The importance of EBV viral loads lies in diagnosis and prognostic evaluation [56]. Increased EBV DNA copies (>10^2.5^ copies/µg) in PB are among the conditions for diagnosis. The EBV DNA load can suggest, to some extent, whether the patient is in an active state [22] and is useful for predicting outcomes [50]. A 2021 study indicated that the median EBV DNA load in PBMCs of the CAEBV group was 5.2 × 10^5^ (IQR 2.3 × 10^4^-3.7 × 10^6^) copies/10^6^ cells [45]. The diagnostic cutoff value of the EBV viral load in PBMCs for EBV-associated diseases was determined to be 1.6 × 10^4^ copies/10^6^ cells in the same study [45]. Compared with clinical practice, the numerical boundaries of diagnostic criteria are conservative.

A small-scale study demonstrated that the load of EBV DNA in PBMCs can aid in distinguishing CAEBV from IM [57]. Patients with HLH were reported to have more EBV DNA copies than CAEBV [6] or without HLH [22]. However, in a Chinese cohort [45], there was no significant difference in the EBV DNA load between any two groups, the CAEBV, EBV-associated lymphoma/leukemias, and EBV-associated HLH groups. According to the above studies, CAEBV has a significantly higher EBV DNA copies than IM, but there is no evidence of higher copies than other EBV-positive T/NK cell proliferation.

There is controversy over the type of blood specimen best for monitoring the EBV load. In 2012, Kimura et al. [6] reported that the quantity of EBV-DNA in PBMCs was significantly correlated with that in plasma, although EBV-DNA was not detected in the plasma of some patients. Studies have shown that plasma levels of CAEBV are more meaningful than peripheral blood mononuclear cell (PBMC) levels for determining diagnostic specificity, distinguishing disease activation, and predicting co-occurrence with HLH [22,58]. Its advantages in treating ENKTL and several EBV-positive lymphomas have been described [59–61]. The combined EBV DNA load cutoff in PBMCs and positive EBV DNA qualitative detection in plasma (>500 copies/mL) allowed for the differentiation of EBV-associated and non-associated diseases, with a sensitivity and specificity of 80.6 and 96.8%, respectively [45]. Accordingly, EBV-DNA in plasma might be more meaningful than in PBMCs, and it could be better to detect both.

Some restrictions still exist. Moreover, EBV DNA PCR levels often lag behind the clinical response and can remain detectable with high-level DNA for several months. Therefore, for patients with complete resolution of symptoms and sustained normalization of biomarkers of inflammation (i.e. blood counts, ferritin), persistent EBV DNA is permitted with close clinical monitoring [34]. EBV DNA was commonly detected in PBMCs from patients without EBV disease, leading to complexities and challenges in the interpretation of positive results.

###### 1.1.3.5. EBV DNA/RNA or viral protein in specimens

The detection of EBV RNA or viral protein in affected tissues and the determination of EBV-infected cells are two basic criteria for the diagnosis of CAEBV. According to a Japanese study of 108 patients, T cells were more common than NK cells and had a worse prognosis. Among the T-cell type and NK-cell type counts, 59% and 41%, respectively, were found [6].

Detection and determination could be performed at the same time. The methods can be classified into three types. (1) Antibody-conjugated magnetic bead sorting was performed, followed by quantitative PCR or FISH [5]. (2) Flow cytometry (3) Histopathology using *in situ* hybridization for EBV-encoded small RNA (EBER). EBER is produced by cells latently infected by the EBV virus, and EBER can be transcribed in large quantities after the EBV infects host cells. This noncoding RNA has a very stable secondary structure and is therefore not easily degraded. EBER detection can be performed using fluorescence *in situ* hybridization (FISH), which labels cells for binding to EBER fluorescent probes by applying fluorescent nucleic acid probes for flow cytometry. FISH can also be used to directly detect the cell phenotype associated with EBV in the periphery or tissue. However, due to the low brightness and uneven distribution of FISH fluorescence, the fluorescence gradually quenches after 24 h, and EBER-negative cells can exhibit nonspecific fluorescence, which exacerbates certain difficulties in reading the fluorescence. *In situ* hybridization (ISH) combined with immunofluorescence (IF) has advantages in the detection of EBER. *In situ* hybridization IF of cells subjected to two antibody incubations after ISH increased the uniformity and brightness of the fluorescence in the nucleus of EBER-positive cells, and the persistence of the fluorescence was greater than that of fluorescent ISH. Pathology specimens are traditionally used. The Japanese criteria explicitly allow the detection of EBV infection in separated T or NK cells from PB. A flow-FISH cytometry assay that detects cells expressing EBERs was published, allowing rapid identification of EBV-infected cells among PBMCs [62]. The detection of proteins such as EBNAs, LPMs, and BZLF1 usually relies on immunostaining and western blotting. Determination of EBV-infected cell types among PBMCs is a valuable tool for the differential diagnosis of EBV + hematological diseases [5]. It is worth mentioning that the use of histopathology to determine the type of infected cell is sometimes different from that used for peripheral blood analysis. Moreover, the explanation and solution remain controversial.

##### 1.1.3.6. Pathology

Histopathological analyses of affected tissues are not specific to CAEBV. Pathological performance of CAEBV and comparisons have been reported in several cases and reviews [43,44,63], and CAEBV usually shows nondestructive reactive inflammatory lesions [64].

The time needed for pathological analysis and overlap of morphologic and immunophenotypic features with those of other methods restrict its application in treating CAEBV^33^. Only 15% of all the patients were successfully diagnosed with CAEBV by histology [19]. The immunohistochemical single-stain technique does not distinguish well between tumor cells and inflammatory cells, and other confounding factors may bias the results [65]. Dojcinov, S. D.et al. [66] summarized and concluded that no changes were suggestive of a malignant LPD. Patients with monoclonal CAEBV of the NK-cell type presented features similar to those of younger ENKTL-ANKL patients, except for more CAEBV patients with juvenile onset [67]. T-cell CAEBV is most often CD4+, which readily differentiates this entity from EBV-HLH and sEBV + TCL. T-cell CAEBV, which is CD8+ and acute in presentation, can overlap with EBV-HLH and sEBV + TCL. NK-cell CAEBV can overlap with EBV-HLH and ANKL. In addition, some of them potentially progress to EBV + T/NK-cell lymphoma.

The diagnosis of NK cell neoplasms requires the integration of clinical presentation, morphology, immunophenotype, and genotype. The expression of at least 1 NK cell marker (CD56, CD16, or CD57); lack of expression of sCD3, B-cell antigens (CD19 and CD20), MPO, and other lineage markers; and/or TCR and Ig genes in the germline configuration in tumors are essential.

Biopsies included lymph nodes, spleen, liver, and bone marrow. Half of the bone marrow biopsy specimens in a single-center study [36] showed no apparent abnormalities. In a case [24], systemic EBV infection involved the myocardium, pericardium, bone marrow, liver, and abdominal wall. In the pericardium, dense infiltrates of mononuclear cells surrounding and infiltrating the vessels were observed, and endomyocardial biopsies were ultimately obtained.

###### 1.1.3.7. Morphology

Histologically, lymph node biopsy reveals a preserved nodal structure, paracortical hyperplasia, and an enlarged lymphoid follicle, occasionally accompanied by epithelioid granuloma and monocytoid B-cell infiltration [26,63]. On cytology, lymphocytes typically bland and mimic chronic inflammatory infiltrates in extranodal sites [26]. Few abnormal lymphocytes with increased cytoplasm and thicker particles were observed in some patients [68]. Lymphocytes infiltrate invaded tissues and are distributed mainly in the lymph node paracortical area, liver sinus, spleen red pulp area, and bone marrow cavity. The cells varied in size; atypia was not apparent, and the number of cells changed. Studies have shown that lymphocyte infiltration is closely related to tissue damage [36].

###### 1.1.3.8. Immunohistology

Among the infected lymphocyte subsets are primarily increased CD4+ T cells, occasionally CD8+ T cells, and CD56+ NK cells [20]. Fewer patients have shown that the dominant EBV-infected cell population is CD8+ T cells [24].

The cells exhibited a Latency I/II viral gene expression profile: EBER+, EBNA1 ±, LMP1, and LMP2a/TR [69]. These T cells were CD3+, granzyme B+, and TIA-1+^36^; no cells expressed the EBV latent membrane protein or EBNA-2 [63].

An analogous dichotomy of CD4+ T cells versus CD8+ T cells is not observed in the NK-cell compartment, rendering distinguishing between systemic NK-cell CAEBV, EBV-HLH with NK-cell expansion and ANKL more challenging [8,44].

### 1.4. Other tests

#### 1.4.1. Chromosomal aberrations and gene mutations

However, studies on the cytogenetic profiles of CAEBV are limited and require further investigation. In 108 CAEBV patients [6], chromosomal aberrations were detected at diagnosis in 6 patients, whereas an additional 6 patients later developed chromosomal aberrations during their clinical course.

The chromosomal aberration classes of ANKL and ENKTL overlap. However, the frequency is different. A study [70] involving array-based comparative genomic hybridization (CGH) revealed that the recurrent region characteristics of the ANKL group compared with those of the ENKTL group included a gain of 1q and loss of 7p15.1-p22.3 and 17p13.1. In ENKTL, the most common cause is a loss of 6q21–6q25 [70,71]. EBV + T-NHL often manifests without cytogenetic abnormalities; therefore, its absence fails to provide definitive significance.

The significance of these gene mutations in CAEBV is likely the same as that of chromosomal aberrations. Gene sequencing may be helpful for the risk stratification of CAEBV. CAEBV can develop based on underlying genetic defects [72–75], so mutations in CAEBV may portend a poor prognosis and etiological background [76,77].

Mutations in SH2D1A, BIRC4, ITK, CD27, MAGT1, CORO1A, and LRBA are associated with severe CAEBV, affecting T and NK cell function while failing to control EBV infection [74]. Concomitant PIK3CD and TNFRSF9 deficiencies cause chronic active EBV infection of T cells [78]. The systematic mutation study in patients with systemic CAEBV was performed by Okuno et al. [79]. Mutations in DDX3X, KMT2D, BCOR/BCORL1, KDM6A, and TET2 have been reported in patients with CAEBV [47]. Among these factors, DDX3X mutation, a marker of poor prognosis, is considered to play a major pathogenic role. Together with POLH and APC mutations, EVC/EVC2 mutations may be a genetic predisposition to CAEBV.

CAEBV shares some mutations with other T/NK-cell proliferation. The mutation of the SH2D1A gene in CAEBV and the XIAP gene was reported [80]. We elucidated the signaling pathways involved in T/NK-cell proliferation [76]. Mutations were detected in nearly or more than half of the patients with EBV + HLH, CAEBV-T/NK, or ENTKL and in 20–30% of the patients with ANKL^77^.

The characteristics of EBV-T/NK-LPD patients are often related to vesicle trafficking genes, such as UNC13D, LYST, ITK, and PRF1 [77]. Mutations in PRF1, STXBP2, and UNC13D are associated with hemophagocytic lymphohistiocytosis [34]. A mutation in STX11 has also been shown to disrupt cytotoxic NK and T-cell functions. In sEBV + TCL, KMT2D was the most frequently mutated gene, followed by MFHAS1, STAT3, EP300, ITPKB, DDX3X, NOTCH1, and NOTCH2 [81]. In ENKTL, the most common mutations involved BCOR, DDX3X, TP53, MGA, STAT3, STAT5b, JAK3, FAS [70,82], KMT2D, TET2, and EP300 [81]; MGA; NOTCH3; EPHA1; PTPRQ; PTPRK; GNAQ; ARID1A; ASXL1; and MLL2 ASXL3 [71,83]. According to the findings of Li-Min Gao et al. [81], mutations in JAK-STAT, TP53, TET2, CREBBP, and MLL2 are frequent in ANKL [84]. In addition, high mutation rates of STAT3, KMT2D, DDX3X, NOTCH1 and TET2 were detected in patients with ENKTL, ANKL and EBV + TL.

##### 1.4.2. 18f-FDG PET/CT

18F-FDG PET/CT is sometimes helpful for identifying the possible trigger (infection or malignant disease) and the extent of secondary HLH. However, PET/CT alone is not sufficient for making a correct differential diagnosis [85]. In initial clinical evaluation, PET/CT is recommended because it is useful for response monitoring and prognostication [86]. PET/CT has been used in the diagnosis and therapy response evaluation of EBV infection-related disorders and has advantages compared with anatomical imaging methods [85,87,88].

There are only cases in which CAEBV was detected *via* PET-CT. Toriihara, A. et al. [89] presented PET/CT images of Epstein–Barr virus-related lymphoid neoplasms and commented on the PET/CT characteristics of CAEBV: despite physical examination revealing the presence of lymphadenopathy and hepatosplenomegaly, these involved organs did not show elevated FDG uptake. Therefore, meaningful discoveries cannot be found [68]. Related symptoms should be taken into account at the same time as diagnosis.

However, in some CAEBV case reports, the determinant findings involved PET-CT scans facilitating targeted biopsy with conclusive histopathological analysis [90,91]. In addition to abnormal activity in the lymph nodes, liver, or spleen, heterogeneous 18 F-FDG uptake associated with mild swelling in multiple skeletal muscle groups may also indicate an active state [91]. In CAEBV enteritis, PET/CT revealed diffuse FDG uptake in the small intestine [92]. In CAEBV-T patients with HLH, hypermetabolic activity was observed in multiple lymph nodes and the spleen [88]. NK/T-cell lymphoma cells are FDG-avid, particularly those of ENKTL, so PET/CT is now considered a standard imaging modality for this lymphoma [60,89].

### 1.5. Aberrant expression of immune cell markers on FCM and analysis

Flow cytometry can be used to obtain as many targeted cells as possible quickly and to perform comprehensive and efficient analysis of cell size, cytoplasmic granularity, and differentiation antigens. FCM in EBV + T/NK LPD patients includes mainly antibodies and clonality evaluation [93,94].

With peripheral blood or bone marrow flow cytometry analysis, test results can be obtained easily and promptly. PB could be used as an alternative to BM for the diagnosis and screening of NK cell tumors [93]. In some cases, the aberrant T/NK-cell population represents <5% of cells in the bone marrow, and these subtle changes may be detected *via* flow cytometry analyses only. This analysis can be performed in any standard laboratory and does not require a large number of samples. Like other laboratory examinations, the assay can be repeated many times.

Flow cytometry (FCM) has been established as a useful tool for the diagnosis, prognosis, and follow-up of some T and NK-cell entities [93–95]. Although good at exploring the status of NK/T cells holistically, its application to CAEBV has been limited because of the presence of overlapping antibodies and a lack of comprehensive analysis studies. FCM is now mostly used to exclude lymphoma or leukemia in CAEBV patients. In an African Systemic CAEBV case report, FCM helped exclude the diagnosis of T-cell lymphoma or lymphoblastic leukemia [90]. The NK cell perforin expression, NK cell degranulation, NK cell cytotoxicity assay, and NK cell interferon-gamma production are used in HLH patients. Moreover, NK cell perforin expression and degranulation-related NK cell cytotoxicity are normal in most secondary HLH patients and usually abnormal in F-HLH patients [96].

Current advances in flow cytometry for the diagnosis of ANKL and ENKTL should further increase the sample size and summarize and verify additional characteristics, including CAEBV. At present, there has been progress in the flow cytometry analysis of EBV-positive NK/T-cell tumors, but CAEBV often progresses to ANKL and ENKL. At present, there are few flow cytometry studies on NK cell-related proliferative diseases, the sample size is small, and there are no flow cytometry studies on CAEBV and its progression; further research is needed.

#### 1.5.1. Normal and EBV-related immune cell markers

Previous flow cytometry studies have evaluated several markers and cell subsets. Different markers of NK cells are connected with their function. The basic function and immunophenotyping of normal NK/T cells are described below.

Common statistical phenotypes of EBV-LPD include the comprehensive immunophenotypic landscape of NK cells, which includes T-series universal markers (CD2, sCD3, CD4, CD5, CD7, and CD8), NK lineage-specific markers (CD16 and CD56), differentiation markers (CD94 and CD57), activation markers (CD30, CD38, and HLA-DR), functional markers of cytotoxicity (Granzyme B and perforin), proliferation markers (Ki-67), depletion markers (PD-1), clonality markers (KIRs: CD158a, CD158b, CD158e1, and CD158i), and other markers used to determine NK cell abnormalities (CD45RA and CD45RO) [93]. The first human NK cell markers identified were CD16 and CD56 [97,98]. Numerous other NK cell markers have since been identified, leading to a better understanding of NK cell differentiation. CD94 is an NK cell receptor of the C-type lectin family that is reported to be uniformly expressed during clonal NK proliferation and variably expressed in reactive cases [99,100]. Perforin is one of the key elements in the function of cytotoxic T cells. Perforinopathies are a group of perforin-deficient hyperinflammatory disorders. This results in the immune system’s failure to fight against and kill virus-infected or altered cells. Granzyme B is critical for cytotoxic lymphocyte-triggered apoptosis [101]. PD-L1 expression, quantified using immunohistochemistry assays, is currently the most widely validated, used, and accepted biomarker for guiding the selection of patients to receive anti-PD-1 or anti-PD-L1 antibodies [102]. To a high extent, the cytotoxic activity of NK cells is regulated by signals from killer immunoglobulin-like receptors (KIRs) [103]. NK cells are cytolytic cells that target tumor cells and bacteria- or virus-infected cells.

NK cells and T cells share a common ontogeny and express T-lineage–associated antigens, including CD2 and CD7. In contrast to T cells, NK cells are negative for surface CD3 but express cytoplasmic CD3 e (e). NK cells also express “NK-associated” antigens, including CD16, CD56, and CD57, with CD56 being the most consistently expressed. Molecularly, T-cell receptor (TCR) genes are in a germline configuration [30].

NK cells are divided into different subsets according to their development and marker expression. *In vitro* studies have identified three subsets of NK cells [104]: NK cell precursors (CD117-CD562, KIR2 [Killer Cell Immunoglobulin Like Receptor], CD162, and CD2441); immature NK cells (CD117 dim, CD561, KIR2, and CD162); and mature NK cells (CD117 dim, CD561, KIR1, and CD161) [104]. The existence of a CD56bright, CD16dim immunoregulatory subset and a CD56dim, CD16+ cytotoxic NK cell subset has been demonstrated [105,106] to be two distinct functional subsets in healthy people. The former is found mainly in secondary lymphoid tissues and comprises 10% of PB NK cells. The CD56bright subset has greater proliferative potential and cytokine-producing function and may be responsible for the ANKL cytokine storm and hemophilic syndrome in some cases of ENKL. The CD56dim population has greater cytolytic activity than the CD56hi population [104,106], which can be demonstrated by the expression of perforated proteins [107–110]. Between CD56bright and CD56dim (CD94/NKG2A–, CD62L–, or CD57+) NK cells, there are CD56dim NK cell subsets (CD94/NKG2A+, CD62L+, or CD57–), which can both proliferate in response to cytokines and activating receptors [111]. The CD56bri NK cell subsets (NKG2A+, CD94+, CD54+, CD62L-) are reported to possibly restrict EBV-induced transformation of B cells [112]. The studies above suggest that EBV infection and systemic CAEBV may affect CD56bright and CD56dim NK cell subsets. It is meaningful to detect EBV activation and inflammation by detecting NK cell-related markers (CD56, CD94/NKG2A, CD62L, CD57, CD54, etc.) at an early stage. The proportions of different NK cell subsets have been shown to have prognostic significance in patients with myeloid malignancies, indicating their importance in the immune surveillance of cancer [106].

These altered NK cell profiles may respond differently to NK-mediated immunotherapies, infections, or vaccines depending on which cytotoxic mechanisms are involved [113].

Jiang et al. reported that normal and reactive NK cells express CD2, CD7, CD11c, CD16, and CD56. A few NK cells did not express CD16 or CD56 in some patients. A small portion of the NK cells did not express CD2 in any of the patients. CD8 and CD57 were partially positive. CD3 and CD4 were not expressed by any of the NK cells [114]. Qi yao Pu et al.^93^divided NK cells into two subsets. The CD56+ subpopulation exhibited characteristics of CD2 + bri/CD7 + bri in addition to not expressing KIRs. The immunophenotype of NK cells largely overlaps with that of T cells. The expansion of dysfunctional CD56-negative NK cells in CMV + EBV + elderly individuals suggested that these cells may infect cellular immunity [115].

It is difficult to determine whether the infection causes changes in immune function or monoclonal tumor cells. Moreover, the markers analyzed by many centers are limited. TCRvβ can be used to determine T-cell clonality. However, reactive proliferation can also occur in monoclonal T cells. NK cells lack a uniquely rearranged antigen receptor gene or a single defining immunophenotypic attribute. A common surrogate for the clonality of NK cells is flow cytometry immunophenotyping of the KIR family of receptors. The expansion of an NK cell clone can be assessed by analyzing the distribution of KIR expression. The aberrant expression of KIRs includes restriction of CD158a, CD158b, or CD158e.

Furthermore, there is considerable functional and phenotypic overlap between NK cells and cytotoxic T cells, and the latter is induced by cellular activation to express many NK-associated antigens. A comprehensive approach to NK-cell immunophenotyping, including the evaluation of NKRs, can enable the distinction of abnormal NK-cell expansions from cytotoxic T cells and reactive NK-cell populations [99].

#### 1.5.2. Peripheral blood lymphocyte counts and subsets

The peripheral blood lymphocyte counts and subsets were subjected to routine immunophenotypic analysis. Although the conclusions are not consistent, an imbalance of lymphocyte subsets and immune dysfunction can be found in CAEBV patients [18,49].

Previous research has shown that NK cell numbers do not exhibit regularity. In 2003, a study in Japan revealed that none of the patients had a low percentage of NK cells [40]. In 2005, in a study by Kimura et al. 19% of patients had elevated NK cells [39]. In 2009, Lu Gen et al. reported that 34.8% of Chinese pediatric CAEBV patients had low NK cell counts [116]. According to the 2011 Cohen study, 43% of CAEBV patients had low numbers of B cells, 33% had low numbers of NK cells, and 38% had low CD4+ cell counts [7]. In 2018, Ling Luo et al. [117]. reported in an adult-onset CAEBV cohort that the percentage of patients with a low CD19+ B-cell count was much greater than that reported in Japan. The percentage of low NK cells was much greater than that in other reports. The percentage of low CD4+ T cells was much greater than that in other reports. In 2021, Jiancheng Lin et al. [49] reported that, compared with those in the control group, the CAEBV group had lower white blood cell counts, lymphocyte counts, CD3+ cells, CD4+ cells, CD8+ cells, NK cells, B cells, CD4 + CD28+ cells, CD8 + CD28+ cells, naive CD4 + CD62L + CD45RA + cells, and naive CD8 + CD62L + CD45RA + cells but higher CD4 + CD25+ cells, DR + CD8+ cells, CD38 + CD8+ cells, effector-memory CD4 + CD62L-CD45RO + cells and effector-memory CD8 + CD62L-CD45RO + cells (all *p* < 0.05). In 2021, Collins et al. characterized CAEBV and HLH patients as monoclonal populations of discrete EBV-activated T-cell subsets; in some cases, these patients had EBV-activated NK-cell ^18^subsets. It is not clear whether these features are significantly different from those of other EBV-associated NK/T-cell proliferative diseases.

#### 1.5.3. Immunophenotyping of CAEBV

However, few studies have been performed to comprehensively analyze and summarize the features and value of immunophenotyping CAEBV.

Immunophenotyping of CAEBV was performed in a few case reports and studies [68]. A CAEBV-NK patient [68] was subjected to flow cytometry before transferring to ANKL, and his flow cytometry analysis revealed that approximately 34.3% of the cells (of total karyocytes of the bone marrow) expressed CD45, CD2, CD7, CD45RO, CD16dim, CD94, and perforin (18% positive), without CD3, CD4, CD8, CD5, CD45RA, CD57, CD11b, cCD3, Ki-67 (1.7% positive), CD158a/h, CD158e, or CD158b.

Understanding the immunophenotyping characteristics of counterparts can help to differentiate or exclude them. We can start by examining the phenotypic features of these subsets and markers. At present, the main phenotypes that need to be distinguished from CAEBV are listed below. 1) Reactive NK/T-cell phenotype 2) NK/T-cell tumor phenotype. CD8+, CD5dim/negative, HLA-DR high, and specific TCR Vβ T cells can be specific markers for the diagnosis of EBV-HLH [94,118]. Moreover, it can be determined whether the current phenotype is related to neoplastic proliferation or reactive hyperplasia. Increases in CD3+ T cells expressing high levels of HLA-DR and CD45RO + memory CD8+ T cells are the characteristic findings in acute IM [119] patients with HV and show activation and expansion of EBV-infected γδ T cells [120].

Abnormal T-cell immunophenotyping lack description. The immunophenotypes of the NK cells are summarized in Table 2. The immunophenotypes of ANKL and ENKL overlap, and their similarities can be summarized together [93,121]: larger forward scatter (FSC), usually CD7-. The CD56+ bright, CD94 + bright, CD26+, CD57- HLA-DR+, Granzyme B + het/-, Perforin + het/-, Ki-67+, PD-1+ or CD30+ and abnormal expression patterns of CD45RA and CD45RO are shown. Their respective characteristics are as follows: ENKTL: CD16-/+dim; most cases are sCD3 − [114,122,123]; absence of CD56 is allowed [122]; CD8-; KIRs-/+ [93]; ANKL [124]: CD16 is more frequently present [27,125]; sCD3- and cCD3+; KIR- [93]; FASL, frequently positive [125]; and CD56dim cases are reported [126]. One study tested the marker depletion marker PD-1 [93] and failed to find regularity. CD11, CD25, CD26, CD27, CD28, and NKB1 were tested in only a few studies [121]. Studies with a small number of samples have not been performed [127].

**Table 2.** Immunophenotype of cells concerned.

A. T-series universal markers (CD2, sCD3, cCD3, CD4, CD5, CD7, and CD8)										
Entity	Year	n	Country	Abnormal NK cells percentages	Immunophenotype					
					CD2	sCD3	CD4	CD5	CD7	CD8
ANKL	2013	43 [114]	China	0.06–76 %	95% +++	/	/	98% -	(47 ± 44)% -	98% -
	2014	29 [124]	China	0.8%-87.7%	97% +++	93% -	97% -	–	48% loss	71% loss
	2022	14 [93]	China	/	+	–	–	–	-/+ het	-/dim
ENKTL	2013	11 [114]	China	0.06–76 %	+++	/	/	/	(45 ± 39)% -	0%
	2022	23 [93]	China	/	+	–	-/rarely	–	-/+het	–
ANKL(*n* = 2) ENKTL(*n* = 10)	2015	12 [121]	Portugal	/	83%	0%	0%	0%	42%	22%

**Table:** 

B. NK lineage-specific markers (CD16, CD56), differentiation markers (CD94, CD57, CD161),								
Entity	Year	n	Country	Immunophenotype				
				CD16	CD56	CD94	CD57	CD161
ANKL	2013	43 [114]	China	(27 ± 34)% −	88% +++	/	0%	/
	2014	29 [124]	China	67% loss	+++	79% +++	–	71% loss
	2022	14 [93]	China	+dim/−	bri	bri	–	/
ENKTL	2013	11 [114]	China	(14 ± 13)% −	+++	/	0%	/
	2018	12 [123]	Singapore	41% +/ dim	75% bright	/	8% +/ dim	/
	2022	23 [93]	China	–	bri	bri	-/+het	/
ANKL(*n* = 2) ENKTL(*n* = 10)	2015	12 [121]	Portugal	10%	>90%	>85%	0%	31%

**Table:** 

C. Activation markers (CD30, CD38, HLA-DR), functional markers of cytotoxicity (Granzyme B and Perforin), proliferation markers (Ki-67),									
Entity	Year	n	Country	Immunophenotype					
				CD30	CD38	HLA-DR	Granzyme B	Perforin	Ki67
ANKL	2013	43 [114]	China	98% −	/	/	/	(21 ± 11)%	/
	2014	29 [124]	China	/	/	/	/	/	typically > 40%
	2022	14 [93]	China	–	+	+/−	+het/−	+het/−	+/−
ENKTL	2013	11 [114]	China	/	/	/	/	(19 ± 12)%	/
	2022	23 [93]	China	–	+/bri	+/−	+het/−	+het/−	/
ANKL(*n* = 2) ENKTL(*n* = 10)	2015	12 [121]	Portugal	/	80%	>50%	/	/	/

**Table:** 

D. Clonality markers (KIRs: CD158a, CD158b, CD158e1, CD158i); Other markers to determine NK cell abnormalities: leukocyte common antigen CD45, CD45RA, CD45RO, TCRs								
Entity	Year	n	Country	Immunophenotype				
				CD158a	CD45	CD45RA	CD45RO	TCR
ANKL	2014	29 [124]	China	7% restricted KIR isoform	52% stronger than normal	/	/	–
	2022	14 [93]	China	/	/	+/-	-/+	/
ENTKL	2022	23^9^ 3	China	-/+het	/	+/-	-/+	/
ANKL(*n* = 2) ENKTL(*n* = 10)	2015	12 [121]	Portugal	0%	100%	>70%	>85%	0%

Several factors contribute to difficulties in recognizing abnormal NK cell expansions and distinguishing them from cytotoxic T cells in the clinical laboratory. A comprehensive approach to NK cell immunophenotyping, including the evaluation of KIRs, can enable the distinction of abnormal NK cell expansions from cytotoxic T cells and reactive NK cell populations [99]. Unlike T cells, however, NK cells lack a uniquely rearranged antigen receptor gene or a single defining immunophenotypic attribute. Furthermore, there is considerable functional and phenotypic overlap between NK cells and cytotoxic T cells, and the latter is induced by cellular activation to express many NK-associated antigens.

The immunophenotype of NK cells in NK cell lymphoproliferative disorders largely overlaps, and for a definitive diagnosis, it is necessary to correlate flow cytometric findings with the morphology, involved anatomic sites, and clinical presentation. In clinical practice, certain immune stimuli can lead to an overlap between the immune phenotype of reactive NK cells and that of neoplastic NK cells. Distinguishing Reactive NK lymphocytosis (RNKL) cells with an altered immunophenotype from tumorigenic NK cells is extremely challenging.

The description of phenotypes *via* CAEBV flow cytometry is valuable for the differential diagnosis of CAEBV. FCM could differentiate disease groups by clustering plots. Qiyao Pu et al. [93] found that CD5, CD16, CD56, CD57, CD94, CD45RA, CD45RO, HLA-DR, KIRs, Granzyme B, Perforin, and Ki-67 were differentially distributed among the RNKL, CLPD-NK, ANKL, and ENKTL groups. In Jiang et al. [114] study, FCM detected some cases earlier than immunohistochemistry did. In the study by Lima et al. [128] found that FCM detected a high proportion of ANKL and ENKTL patients. In the studies above, the FCM results were in agreement with those of the IHC studies performed on tissue biopsies, at least for the markers more frequently used to support the diagnosis.

By adding additional data to the CAEBV cohort, we might have identified differentially expressed markers in CAEBV patients. Immunophenotyping approaches using antibodies against traditional and novel T- and NK-associated antigens can be used to evaluate these cell types and diagnose NK-cell and cytotoxic T-cell disorders in the clinical laboratory setting [99]. The identification of the phenotype of abnormal NK cells in CAEBV may help elucidate the underlying mechanism and aid in diagnosis. CAEBV can cause inflammation or neoplastic disease. It may be characterized by both reactive hyperplasia and neoplastic hyperplasia. Moreover, differences in marker expression, forward scatter (FSC) and/or side scatter (SSC), and the monoclonality of mature NK cells suggest differences in NK cell tumors and other diseases. A combination of clinical features and patient outcomes can aid in the diagnosis and early identification of ANKL or ENKTL. The FCM characteristics of these patients indicated that different types of neoplasms might be derived from different NK cell subtypes. FCM characteristics might also indicate its source. By comprehensively analyze the CAEBV phenotype, we may be able to identify the relatively unique phenotypic characteristics of CAEBV, which could be used to help diagnose this disease.

The description of phenotypes *via* CAEBV flow cytometry is expected to be explored for prognostic guidance. According to previous studies, abnormal expression of some antibodies is thought to be associated with prognosis. CD56 is a nerve cell adhesion molecule [98], and its strong expression is associated with tumor invasion and poor prognosis [129,130]. Elevated FSC is also useful evidence for tumor cells. To some extent, the FSC can also reflect patient prognosis. The prognosis of ANKL patients with large FSC is poor, while the prognosis of patients with basically normal FSC is relatively good. On the other hand, although the CD56 MFI and FSC are not significantly different between ENKL and ANKL patients, the prognosis for both is poor [131]. It has also been reported that CD7 and CD45RO expression is related to a poor prognosis in ENKTL patients, while those with positive KIRs have a better prognosis [93]. PD-L1 levels in tumor tissues may affect the outcomes of ENKTL patients [132].

The current classification of CAEBV is based on pathology and clonality. Characteristic tumors were mainly excluded by pathology. Current research on prognostic factors has revealed a small number of clinical features, such as advanced age. However, the relationship between flow cytometry characteristics and the prognosis of CAEBV patients has not been studied.

#### 1.5.4. DNA or RNA flow cytometry

DNA/RNA flow cytometry techniques are not widely used. We now introduce two of them. The first is used to determine the median DNA index and cell cycle status of neoplastic NK cells [128]. A hyperploid DNA content can be in agreement with the complex cytogenetic abnormalities observed in NK cell tumors. The high percentage of cells in S-phase might also be related to the IHC results for the Ki67 nuclear proliferation marker. The second technique named FlowRNA combines surface marker staining with *in situ* hybridization for EBER RNAs [18, 62]. FlowRNA detected disease relapse arising from re-emergence of the original EBER + CD4+ T-cell clone. This approach enables a thorough examination of the number and function of EBV-infected T/NK cells.

#### 1.5.5. Clonality

It is difficult to determine whether the infection causes changes in immune function or monoclonal tumor cells. Moreover, the markers analyzed by many centers are limited. TCRvβ can be used to determine T-cell clonality. However, reactive proliferation can also occur in monoclonal T cells. NK cells lack a uniquely rearranged antigen receptor gene or a single defining immunophenotypic attribute. A common surrogate for the clonality of NK cells is flow cytometry immunophenotyping of the KIR family of receptors. The expansion of an NK cell clone can be assessed by analyzing the distribution of KIR expression. The aberrant expression of KIRs includes restriction of CD158a, CD158b, or CD158e.

## 2. Conclusions

Some diseases related to EBV-LPD have a transformation, crossover, or intersection of clinical manifestations, and it is necessary to reasonably stratify the risk of these diseases and diagnose ANKL, high-risk CAEBV, and NK/T-cell lymphoma on time to the greatest extent possible. It is important to choose the correct inspection method and fully interpret the results. Specific and sensitive clinical criteria are desperately needed to accurately differentiate between CAEBV and other cancers at the time of initial clinical presentation and provide a risk stratification platform for guiding therapy. However, traditional or current testing methods are slow and nonspecific.

For CAEBV patients, tumor treatment methods, ranging from clinical manifestations to EBV load, pathology, flow cytometry, molecular, and genetic information, should be used. Comprehensive analyses of age, clinical manifestations, disease progression rate, pathology, and infected cell type are needed for diagnosis and determination of molecular characteristics. Moreover, pathological features are still relatively accurate, and pathological tissues should be examined as much as possible. The means to develop forecasts are a priority. Age, the presence of HLH, the pathological type (cutaneous), the plasma EBV load (1 × 10^4^ copies/ml), the presence of a panel mutation, the tendency for tumor transformation, the presence of dynamic changes in Ki-67 expression and pathology are prognostic risk factors.

The increase in the percentage of a particular lymphocyte subpopulation may vary depending on the clinical condition. A summary of the immunophenotypic characteristics of patients with chronic active EBV infection can help in diagnosing CAEBV. If we can track the changes in phenotypic characteristics of outcomes, determine the significance of flow cytometry in the diagnosis and prognosis of CAEBV patients, and identify markers or combinations of markers that predict clinical behavior, flow cytometry could aid in the diagnosis and early treatment of high-risk CAEBV patients. The FCM immunophenotype might be an important tool for the classification and treatment of CAEBV, indicating the prognosis and selection of potential therapeutic targets. There is still a need to comprehensively integrate and compare the characteristics of this series of diseases, and an effective risk prediction scoring system still needs to be explored (see Figure 1).

## Data Availability

Data sharing is not applicable to this article as no new data were created or analysed in this study.
